# The fruit and vegetable import pathway for potential invasive pest arrivals

**DOI:** 10.1371/journal.pone.0192280

**Published:** 2018-02-16

**Authors:** Erik Lichtenberg, Lars J. Olson

**Affiliations:** Department of Agricultural and Resource Economics, University of Maryland, College Park, Maryland, United States of America; Chinese Academy of Agricultural Sciences Institute of Plant Protection, CHINA

## Abstract

The expansion of international trade in commodities increases the risk of alien species invasions. Invaders are difficult to detect on introduction, so prevention remains the preferred strategy for managing the threat of invasions. Propagule pressure has been shown to be a good predictor of invasion risk. Most studies to date, however, link potential invasive species arrivals with indirect measures of propagule pressure such as aggregate trade volumes. This paper estimates propagule pressure using data that measure actual arrivals. Specifically, it uses inspection data that covers almost all U.S. fruit and vegetable imports from 2005–2014 to estimate a logit model of the probability of potential invasive species arrival and expected propagule frequencies for 2,240 commodity/country of origin combinations. Clear patterns in the geographic origin and commodity pathways for potential pests are identified. The average probability of arrival is low, approximately 0.03, but is two to ten times higher for some commodities, most notably herbs. We identify commodities with a high number of expected arrivals due to either a large volume of trade, high interception rates, or a combination of both. Seven of the top ten countries of origin for propagule frequency are from the Western Hemisphere and further trade liberalization within the Western Hemisphere is likely to heighten challenges to enforcement of US phytosanitary standards. Patterns in the data can help identify the commodities and countries of origin in greatest need of technical assistance and guide targeting of surveillance for the pathways of greatest phytosanitary concern.

## Introduction

The expansion of international trade in commodities is beneficial in many ways, offering buyers a greater diversity of products and lowering purchase prices of familiar products [1]. At the same time, the global movement of goods enhances the threat of invasions by nonindigenous species that cause economic and ecological harm [2–11]. Expanded trade amplifies the probability of biotic invasions by increasing both the volume of attempted introductions and the diversity of introduced nonindigenous organisms as the diversity of trading partners has [5–6,11–13].

Invaders are difficult to detect in early stages of establishment and difficult to control once firmly established, hence prevention is typically the preferred management strategy [14–16]. Screening imports for potentially harmful nonindigenous organisms is an important component of the safeguarding continuum. Current screening practices involve examining a sample of approximately 2 percent of each shipment, adjusted according to perceived risk and workload [17]. Adjusting sampling effort using quantitative risk assessments has been shown to be both possible and cost effective by providing more consistent risk management, making better use of available resources, and reducing incentives to circumvent phytosanitary safeguards [18–20].

Key components of any risk assessment used to set screening strategy are measures of propagule pressure such as propagule frequency and size (the number of arrivals of nonindigenous pest species, and the number of organisms in each arrival). While estimation of propagule pressure was thought for many years to be too subject to error due to the base rate effect to be reliable for devising guides for decision making (see for example [21]), recent work has shown measures of propagule pressure to be among the best predictors of invasion potential [5,22–27]. Data from the Australian ornamental plant trade indicate that estimates of risk factors—including propagule pressure—measured with current accuracy rates can be used to devise screening strategies that generate positive net benefits in terms of avoided damage from invasive nonindigenous species relative to foregone trade value [18].

Most empirical studies linking invasion risk and propagule pressure relate the presence of alien species to indirect measures of propagule pressure such as historic trade patterns [9,18,24] The inferences drawn from such analyses are suggestive but not definitive. Manufactured commodities only provide indirect pathways for invasion via packaging or hitchhiking in containers or transport. In contrast, trade in agricultural commodities serves as a direct pathway and phytosanitary measures often focus on these commodities. This paper employs data that measure propagule pressure directly using records from surveillance screening of fruit and vegetable imports by the Animal and Plant Health Inspection Service of the US Department of Agriculture (APHIS). The data cover nearly all fresh fruit and vegetable imports to the U.S. over the 10 year period from 2005–2014; approximately 2.8 million individual shipments comprising 139 different fruit and vegetable commodities imported from 64 different countries/regions; a total of 2,240 commodity/region combinations with at least one shipment. They record whether or not an actionable pest was detected in each individual shipment and thus give a direct measure of patterns of potential pest arrivals via the agricultural commodity pathway.

We use a logit model to estimate the predicted probability of arrival of a potential pest as a function of commodity, country/region of origin, season, port of entry, tariff status and shipment volume. We then estimate the annual number of shipments expected to contain at least one actionable organism by these same characteristics as a measure of the number of arriving propagules. Upper and lower bounds of 95 percent confidence intervals for these estimates are calculated using the delta method. The statistical model derives its power from the likelihood of pest interceptions that are commodity specific across regions, reflecting pest-host relationships, or region specific across commodities, reflecting phytosanitary measures and other country specific characteristics. It does not model characteristics that are unique to a specific commodity-region pair. We use the model to identify targets for enhanced surveillance scrutiny and for technical assistance to help exporters and foreign governments improve phytosanitary performance.

## Materials and methods

### Data

We use inspection data maintained by the Animal and Plant Health Inspection Service of the US Department of Agriculture (APHIS) to estimate measures of the frequency of potential pest arrivals to the US through trade in fruits and vegetables. The data, recorded on APHIS/PPQ Forms 264 and 280, encompass the vast majority of fresh fruit and vegetable imports to the U.S. over the 10 federal fiscal years from 2005–2014. They contain information on 2,873,091 shipments comprising 139 different fruit and vegetable commodities imported from 64 different countries/regions, corresponding to a total of 2,240 commodity/region combinations with at least one shipment. Of these, 105,219 shipments were pre-cleared and thus not subject to normal inspection while 396 shipments of grapefruit and 1 shipment of salmonberry had no intercepts. We were unable to classify commodities for 8,219 shipments. Two shipments identified as originating in North Korea were dropped. Thirteen observations were missing the quantity of the commodity imported and 17 observations were missing the port of entry. The sample size for our analysis consists of the remaining 2,759,224 inspected shipments (96% of all shipments entering the US). Actionable organisms were detected in 90,649 or 3.3% of those shipments.

The APHIS 280/264 data include a disposition code that indicates shipments associated with an actionable organism. APHIS determines whether a pest is actionable based on its novelty and known prevalence or distribution within and throughout the United States; its potential harm to U.S. agricultural, environmental, or other resources; and the need to mitigate its pest risk [28]. Many pest intercepts are not identified to the species level and precautionary action may be required if an organism is identified as belonging to a genus or family that includes a regulated pest. The data do not distinguish between actions associated with regulated pests and precautionary actions. A potential pest arrival is defined as the detection in a shipment of at least one actionable organism sufficient to trigger action (e.g., treatment or refusal of entry). Since inspection may cease when an actionable organism is found, the data provide a measure of propagule frequency but not necessarily propagule size [29].

The APHIS 280/264 data also include the date of entry, the port of entry, the name and type of commodity, the shipment’s country of origin, the quantity of the commodity contained in the shipment and whether the commodity was pre-cleared in the country of origin (e.g., grapes in Chile) or inspected at reduced rates under the National Agricultural Release Program (NARP) or its predecessor, the Border Cargo Release program. Some shipments entering under pre-clearance or NARP and some pre-treated shipments are inspected to ensure ongoing compliance with the terms of those programs. Ferrier [30] contains a more detailed description.

We aggregated commodities into groups that correspond to categories identified by the Harmonized Tariff Schedule of the United States (HTS). We created a concordance between the commodity names occurring in the APHIS 280/264 data and categories occurring in the HTS for each year of the sample period. We used that concordance to merge the APHIS 280/264 data with the tariff schedule data for each year at the 8-digit HTS code level associated with unique tariff rate lines. We then truncated the 8-digit HTS codes to the 6 digit International Harmonized Commodity Description and Coding System for a number of commodities with low intercept rates to avoid losing data for 8-digit tariff lines with few or no actionable pest detections. Groups of commodities aggregated at the 6-digit HTS code level included: fresh black beans, lentils, and other legumes; chicory, endive, escarole, and radicchio; currants, gooseberries, and tamarinds; tropical and Chinese fruits; collards, kohlrabi, and rape; nuts; cinnamon, nutmeg, turmeric, and other spices; and fennel, palm hearts, ginseng, horseradish, and various leaves.

Over the 10 year period of the data, the U.S. imported fruits and vegetables from 130 different countries. For each country of origin we distinguish two growing seasons: summer (May through October in the Northern Hemisphere and November through April in the Southern Hemisphere) and winter (November through April in the Northern Hemisphere and May through October in the Southern Hemisphere). The prevalence of potentially harmful nonindigenous organisms in exporting countries and thus infestation rates in import shipments is expected to be greater in summer than winter. Seventy-two countries of origin averaged fewer than 10 shipments per year or had only 1 or 0 intercepts over the entire 10 year period of the sample. These minor trading partners were grouped by geographic proximity into 6 secondary regions: Africa secondary (15 countries), Caribbean secondary (6 countries), Europe secondary (22 countries), Middle East secondary (5 countries), SE Asia secondary (21 countries) and South America secondary (3 countries). This procedure condensed the number of countries to 64 country/regions of origin. Ports of entry were aggregated into 5 regions. All else equal, we expect that inspection of import cargoes—and thus detection of harmful nonindigenous organisms—is greater in regions of entry in which potential damage from invasive pest introductions is greater.

### Statistical analysis

We use a logit model to estimate the predicted probability of arrival of a potential pest as a function of commodity, country/region of origin, season, port of entry, tariff status and shipment volume. We then estimate the annual number of shipments expected to contain at least one actionable organism by these same characteristics. This provides a measure of the number of arriving propagules. Upper and lower bounds of 95 percent confidence intervals for these estimates are calculated using the delta method. We use the model to identify targets for enhanced surveillance scrutiny and for technical assistance to help exporters and foreign governments improve phytosanitary performance. The considerations discussed above suggest that the probability that at least one actionable pest is detected in shipment j, ϕ_j_, conditional on the characteristics of the shipment, should be specified as:

$$\phi_{j}=\Lambda \left(a_{0}+\sum_{n}a_{1n}{Commodity\;Type}_{jn}+\sum_{n}a_{2m}{Origin}_{jm}+\sum_{t}{a_{4t}Season}_{jt}+\sum_{r}a_{5r}{Port}_{jr}+a_{6}{Shipment\;Volume}_{j}+u_{j}\right)$$

where u_j_ represents unobserved factors influencing the presence of one or more actionable pests. This statistical model derives its power from the likelihood of pest interceptions that are commodity specific across regions, reflecting pest-host relationships, or region specific across commodities, reflecting phytosanitary measures and other country specific characteristics. It does not model characteristics that are unique to a specific commodity-region pair.

The estimated coefficients of the logit model are used to calculate predicted probabilities that a potential pest would be detected for every observation in our data set. The average over all observations containing the commodity or region from which that commodity has been imported provides an estimate of the probability of a potential pest detection for each commodity/region combination. Probabilities of arrival by commodity and by region of origin are derived in the same manner. Confidence intervals are estimated using the delta method. The ratio of the predicted probability of an intercept to the overall sample average is used as a measure of the relative likelihood of potential pest arrival for each commodity/region combination.

Estimated coefficients of the logit model are given in the S1 Table of the Supporting Information. An estimate of propagule frequency or the scale of potential pest arrivals is obtained by multiplying the estimated probabilities by the average annual number of shipments of each commodity from each region of origin during each season entering each region of entry over our sample period. The S2 and S3 Tables of the Supporting Information report the estimated probabilities that a single shipment contains a potential pest and the predicted annual number of shipments containing potential pests for all commodities (aggregated across regions of origin, production seasons, tariff status and ports of entry) and regions of origin (aggregated across commodities, ports of entry, production seasons and tariff status), respectively.

## Results

**Fig 1:**
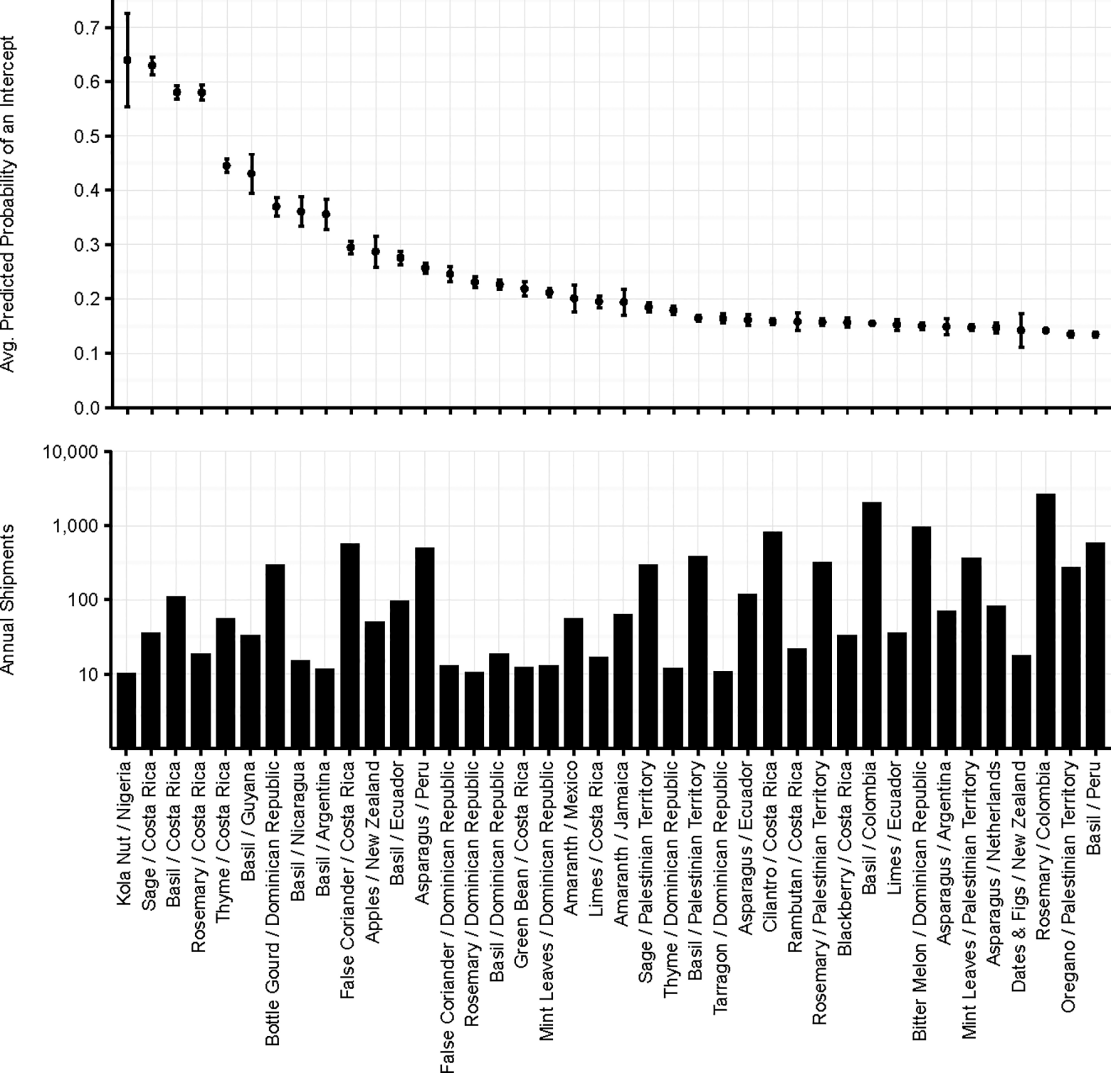
Estimated probability of pest arrivals by commodity/country of origin combination. Estimated probability of at least one potential pest arriving in a shipment for the top 40 commodity/country of origin combinations with at least 100 shipments over 10 years.

Fig 1 depicts the predicted probabilities that at least one actionable organism is intercepted in a shipment for the top 40 commodity/region of origin combinations entering the US that had at least 100 shipments over the 10 years in our sample (i.e., averaged at least 10 shipments per year). Nineteen of these commodity/region of origin combinations have predicted probabilities of potential pest arrival 0.20 or higher, more than six times the sample average of 0.033. All of the top 40 commodity/region combinations entering the US have probabilities of potential pest arrival of at least 0.14 while another 31 have probabilities of arrival between 0.10 and 0.14 (see S2 Table of the Supplementary Information).

**Fig 2:**
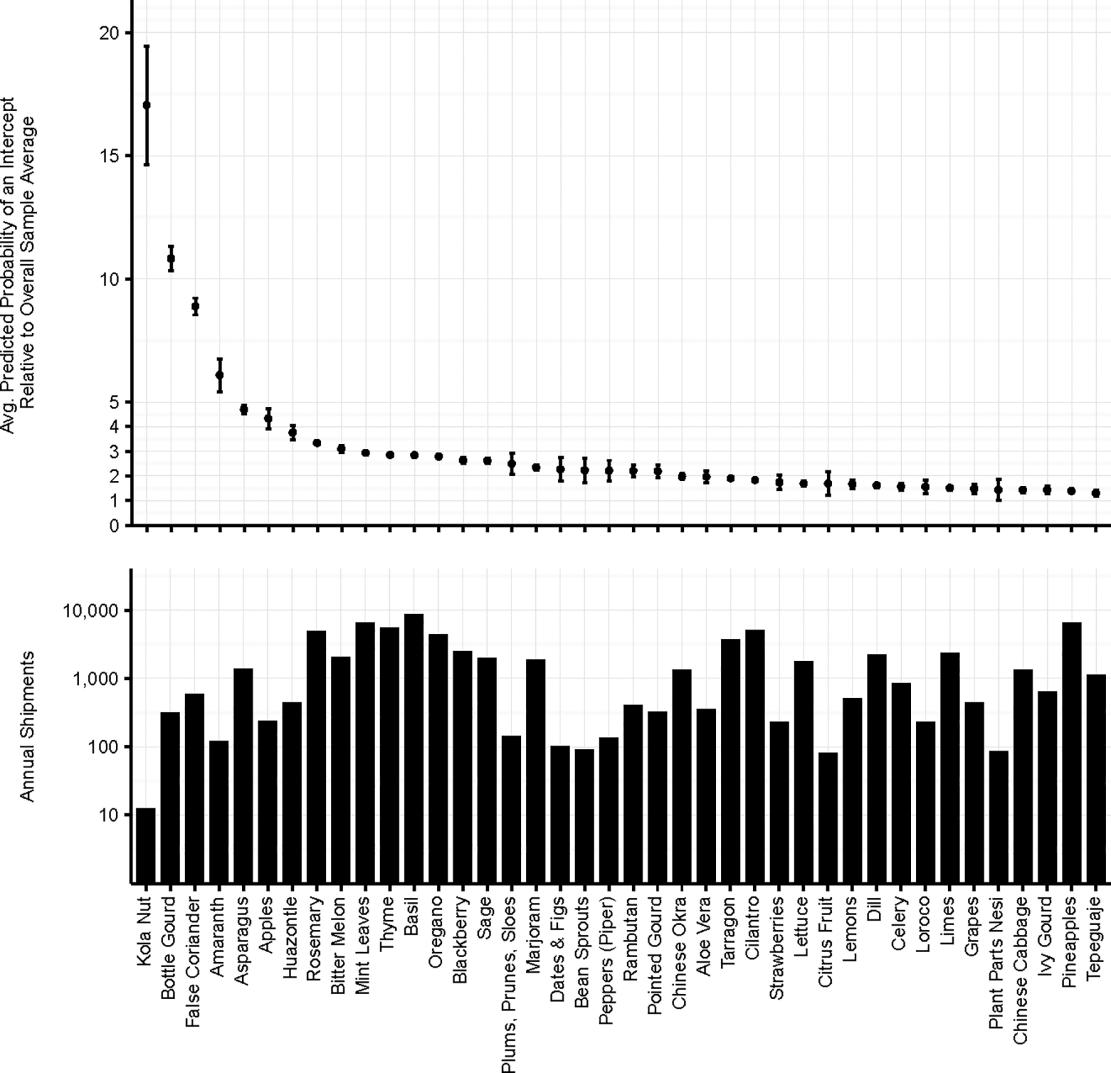
Estimated relative probability of pest arrival by commodity. Estimated probability of at least one potential pest arriving in a shipment relative to the overall sample average for the top 40 commodities.

**Fig 3:**
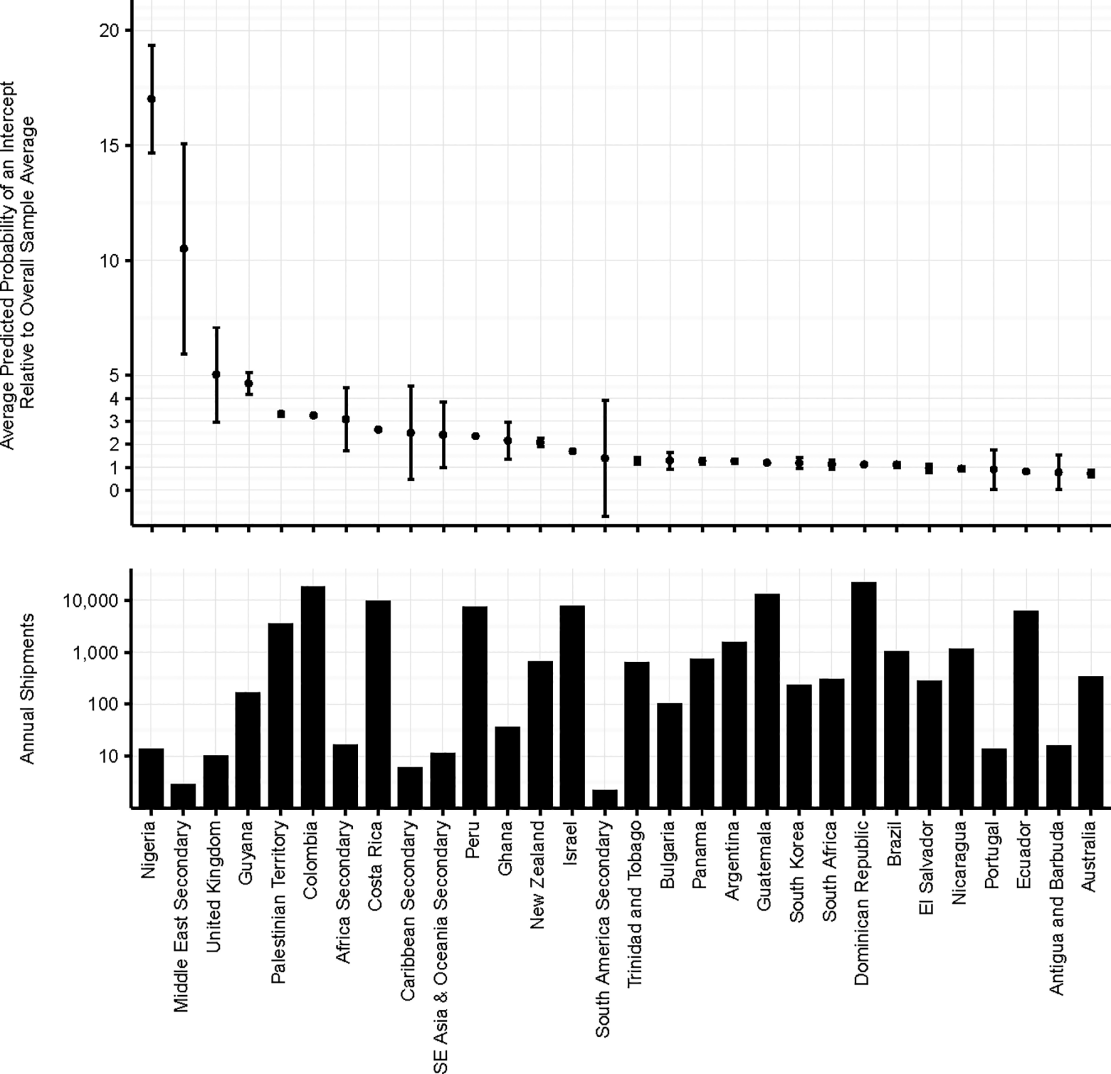
Estimated relative probability of pest arrival by country of origin. Estimated probability of at least one potential pest arriving in a shipment from each country/region of origin relative to the overall sample average.

Probabilities of actionable detections relative to the overall sample average by commodity and region of origin are depicted in Figs 2 and 3, respectively. Among vegetables, squashes (gourds, bitter melon, Chinese okra/luffa), asparagus, peppers and head lettuce have relative probabilities at least half again as large as the overall average. Numerous fresh herbs (false coriander, rosemary, mint leaves, thyme, basil, oregano, marjoram, dill) also have predicted intercept rates at least 50% greater than the overall average. While some vegetables also exhibit high predicted arrival rates, most notably winter vegetables from the Western Hemisphere (tomatoes and lettuce from Central America), vegetables overall account for the bulk of the commodities with the lowest probabilities of potential pest arrival (S2 Table –Supplementary Information).

Nigeria, various countries in the Middle East and the United Kingdom are shipment origins with the highest predicted intercept probabilities, with averages across all commodities, seasons and ports of entry 5–17 times the overall 10-year average (Fig 3). Countries in South and Central America, the Caribbean, in Southeast Asia/Oceania and elsewhere in Africa and the Middle East are the next largest, with averages across all commodities, seasons and ports of entry 2–5 times the overall 10-year average. At the other end of the spectrum, Canada and China are very low probability sources of potential pest arrival, with averages across all commodities, seasons and ports of entry 0.1–0.2 times the overall 10-year average.

**Fig 4:**
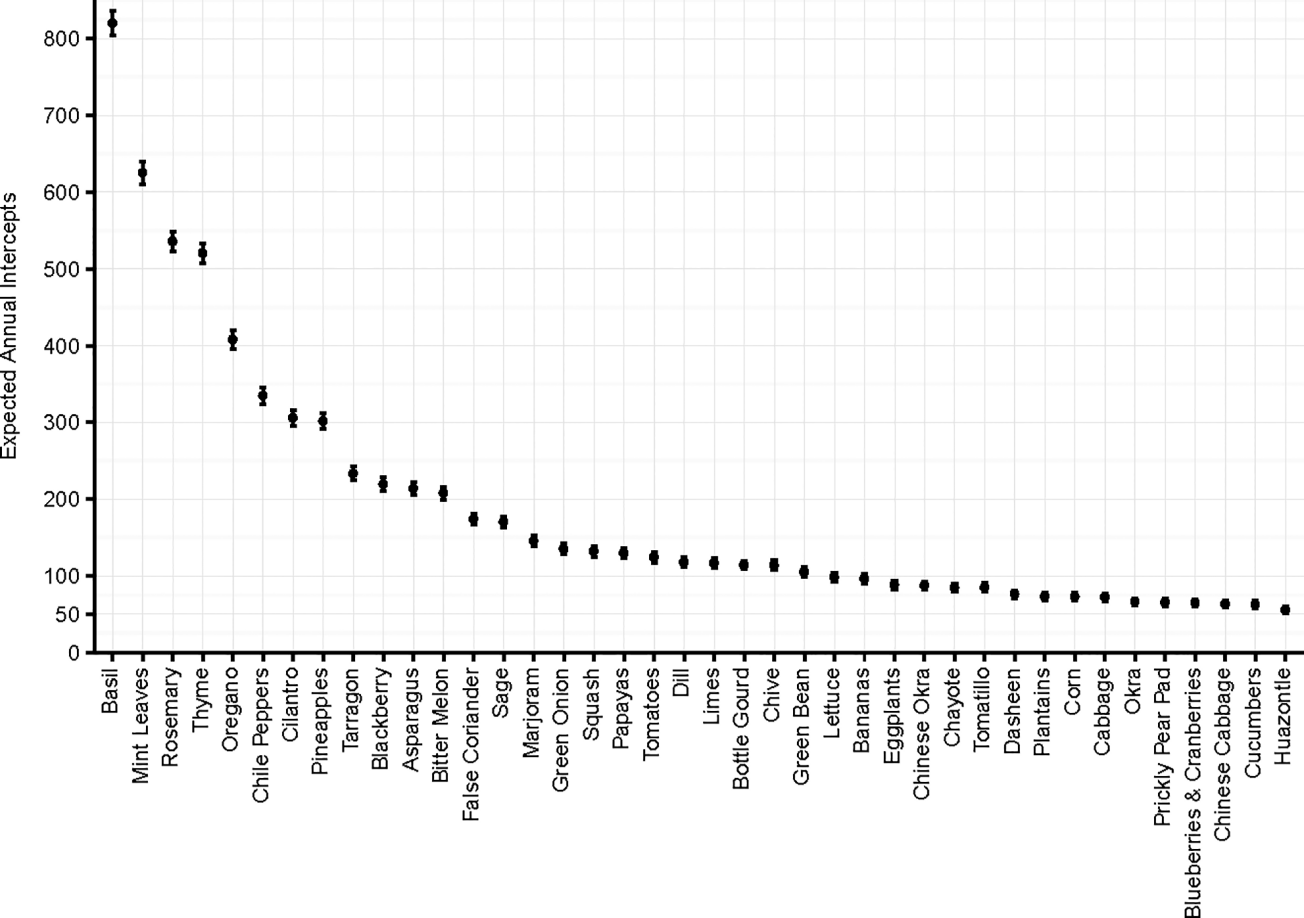
Expected annual invasive pest arrivals, top 40 commodities.

The number of attempted introductions is a better measure of propagule pressure than the probability of arrival in an individual shipment [5, 22–24]. The expected annual number of shipments with a potential pest detection is our second frequency measure. It provides an estimate of the number of instances propagules are expected to arrive each year in shipments of a given commodity or from a given country/region of origin. Fresh herbs are associated with the highest expected propagule arrival frequencies by this measure: basil, mint, rosemary, thyme and oregano account for an expected 410–820 arrivals of potential pests annually while cilantro, tarragon, false coriander, sage, marjoram and dill account for an additional 110–310 expected annual arrivals (Fig 4). Some vegetables with high predicted intercept probabilities also exhibit very high predicted propagule arrival frequencies: chili peppers, asparagus, and squashes (bitter melon, bottle gourd, Chinese okra/luffa) account for expected pest arrivals on the order of 90–340 annually. At the same time, some vegetables with moderate intercept probabilities exert substantial propagule pressure due to large numbers of shipments. For example, green onions, tomatoes, green beans eggplants, cabbage and corn have predicted intercept rates below the overall sample average but account for 70–135 expected actionable pest arrivals annually.

**Fig 5:**
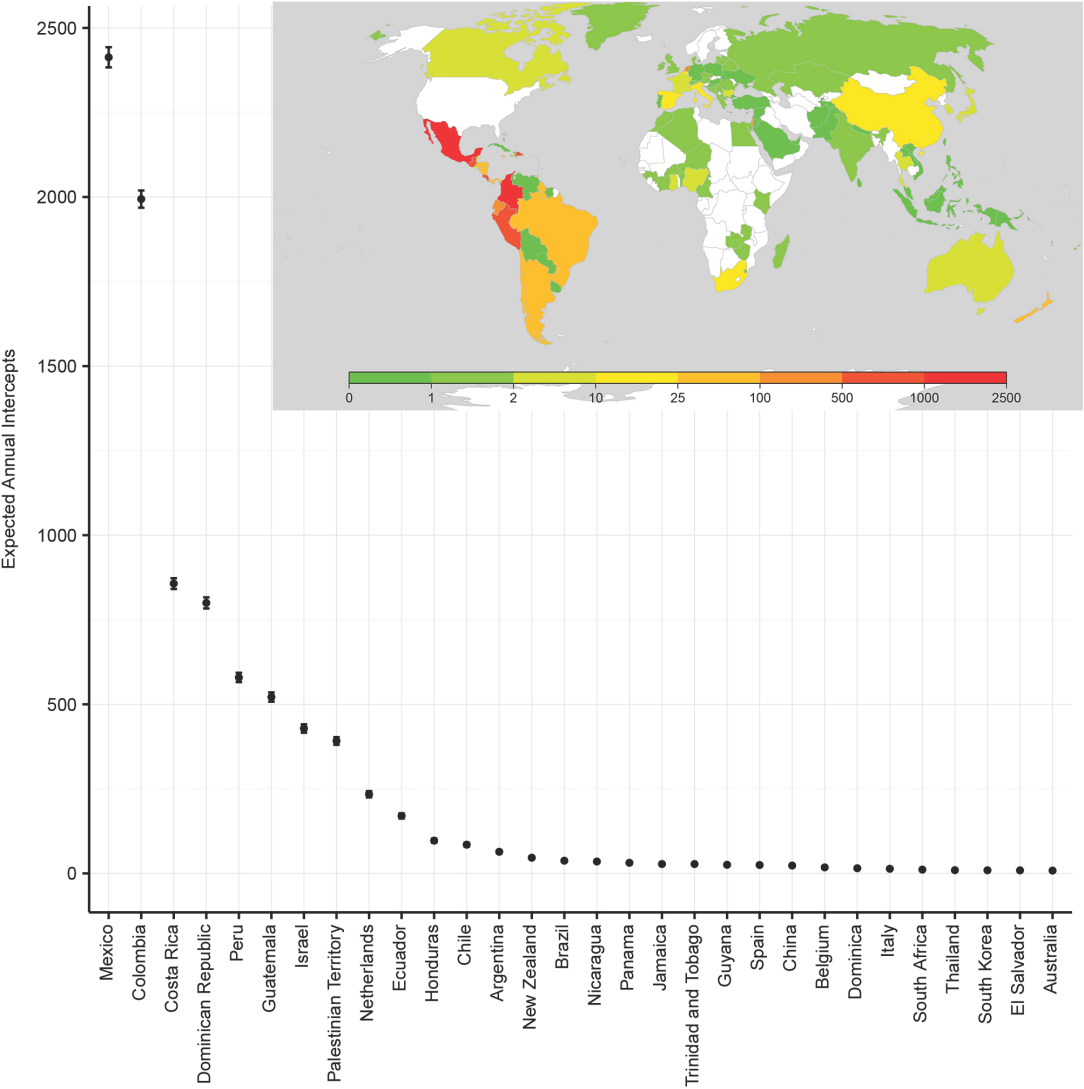
Geographic distribution of expected annual pest arrivals.

As the predominant source of fruit and vegetable imports to the U.S., the New World is the largest source of expected arrivals of potential pests (Fig 5). Mexico alone accounts for 2,400 expected actionable shipments annually while Colombia accounts for almost 2,000. Ten exporting countries are associated with 100 or more expected annual arrivals of potential pests. Seven are located in South America, Central America and the Caribbean; Israel, the Palestinian Territories and the Netherlands make up the remainder. On the other end of the spectrum, Canada, Japan, India, Turkey, Taiwan, most countries in Europe and smaller exporters in South America, the Caribbean and Southeast Asia account for less than 5 expected actionable pest arrivals annually, while Nigeria, Australia, El Salvador, South Korea and Thailand account for less than 10 per year.

The high expected numbers of annual propagule arrivals in fruit and vegetable imports from countries like Colombia, Costa Rica, the Dominican Republic, Guatemala, the Netherlands and Israel arise from a combination of higher than average predicted intercept rates and a large number of shipments, as each country accounts for 4–8% of the total number of observations in our sample (Fig 3). Mexico’s dominance as a trading partner for fruits and vegetables is a primary reason why it is one of the highest sources of actionable pest detections. The overall intercept probability from a single shipment from Mexico is low (averaged across commodities, seasons and ports of entry), but the number of shipments of fresh fruits and vegetables is extremely large—over half the observations in the data come from Mexico. The Netherlands is a key transshipment hub that connects global markets and imports labeled as originating in the Netherlands often originate in other countries. For the United Kingdom and many African countries, the predicted probability of an intercept associated with each shipment is relatively high but the number of shipments is so small (less than half a percent of the total number of observations) that the estimated propagule frequencies from those countries is quite low.

## Discussion

**Fig 6:**
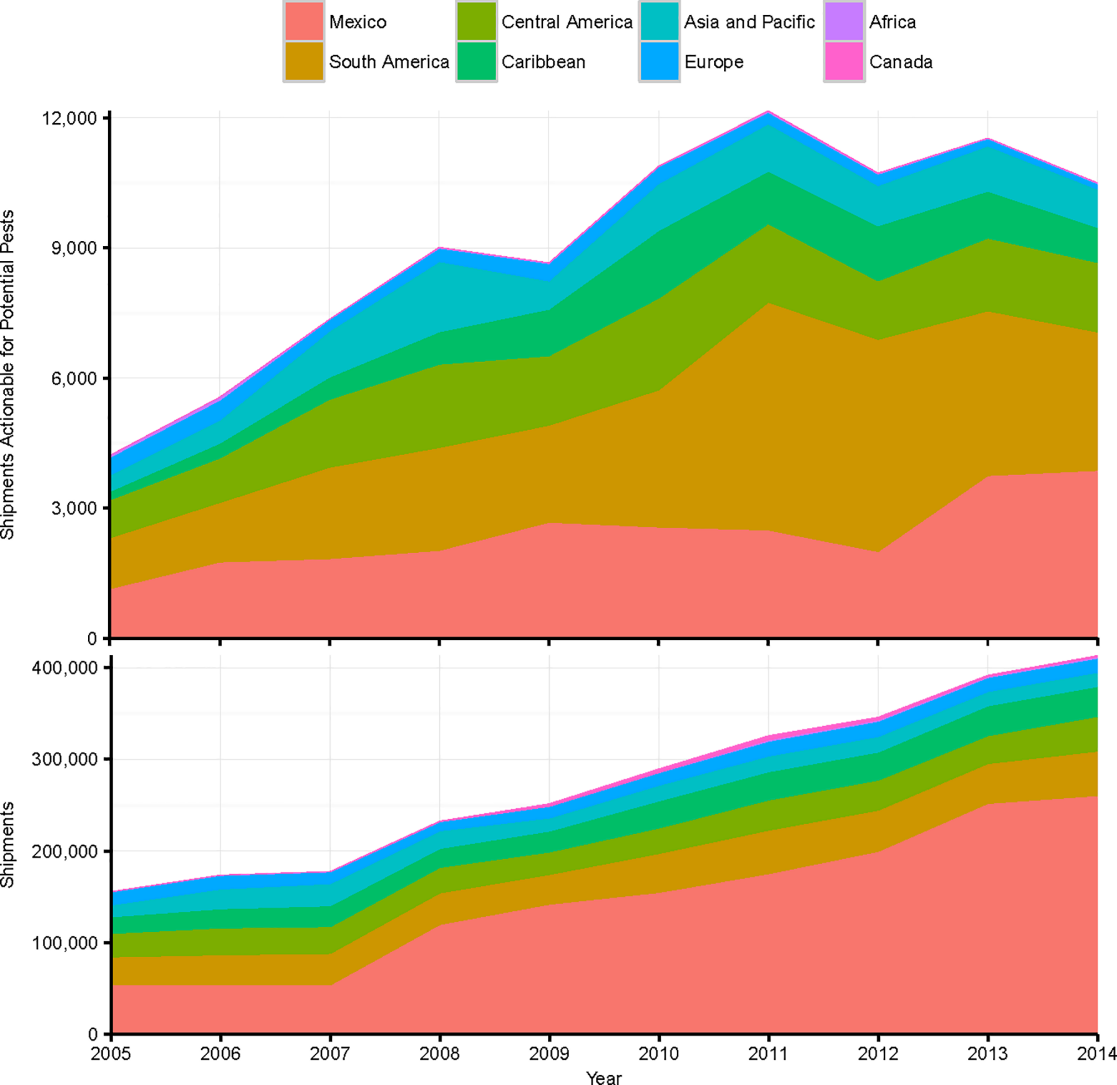
Fruit and vegetable imports: Total and actionable shipments, 2005–2014.

Over the past decade, imports of fruits and vegetables into the US have grown rapidly, so that the US is now a net importer [31]. Imports come primarily from the Western Hemisphere south of the US border (Fig 6). The US has free trade agreements with Mexico and Canada (the North American Free Trade Agreement), Central America (the Central American Free Trade Agreement), Chile, and Peru. Fruit and vegetable imports from other South American countries like Argentina and Brazil have been growing as well and the US grants preferential access to imports of many commodities from the Caribbean under the Caribbean Basin Initiative.

Canada and Mexico are of special interest from a policy perspective because US growers have often cited the risk of pest invasions a reason for continuing to uphold trade barriers with these countries. We find mixed evidence for this contention. The probability of encountering an actionable pest in a shipment from Canada is extremely low, about 0.002; the annual number of predicted arrivals is less than 5, so that Canada accounts for a negligible share of shipments containing potentially invasive pests. We find that Mexican shipments are about half as likely as the average shipment to have an actionable intercept (0.016 versus 0.033), but the expected propagule frequency from Mexico is nevertheless quite high because Mexico accounts for over half of all shipments. Our model predicts that Mexico accounts for 27 percent of the annual average of 9,065 fruit and vegetable import shipments containing actionable organisms.

Among Central and South American countries, Colombia and Costa Rica, the Dominican Republic, Peru and Guatemala are the sources of the largest numbers of expected annual pest arrivals after Mexico (Fig 5). Chile, Argentina, and Brazil also account for large numbers of invasive pest arrivals. Overall, Central and South American countries account for about a fifth of current fruit and vegetable shipments and almost two-fifths of expected annual pest arrivals. The Caribbean has a predicted intercept probability equal to the overall average of 0.033; its frequency of invasive pest arrivals (10 percent of the total annual number of predicted arrivals) is thus proportional to its large share of fruit and vegetable import shipments (9.5 percent of the total).

Our analysis helps to quantify how much the expansion of trade in fruits and vegetables ensuing from these trade liberalization agreements has raised US exposure to invasions of potentially harmful nonindigenous species. Fruit and vegetable imports from Central and South America have relatively high frequencies of actionable pest arrivals, as do imports of many common fruits and winter vegetables from Mexico. Pest intercepts from Central and South America have grown at a faster rate than fruit and vegetable imports (Fig 6). Further trade liberalization within the Western Hemisphere is thus likely to heighten challenges to the enforcement of US phytosanitary standards.

We find a very low incidence of potentially invasive pests in fruit and vegetable exports from China to the US: The predicted probability of a pest arrival is 0.007 and the predicted annual number of shipments containing actionable organisms is only 20. In contrast, Middle Eastern countries have high predicted probabilities of actionable pest detections (Fig 3), which is likely due to high background pest infestation rates coupled with poor sanitation practices. The volume of imports is so small, though, that the predicted number of potential pest arrivals is very low. Our model indicates that any expansion of trade with this region is likely to result in an increase in propagule pressure.

**Fig 7:**
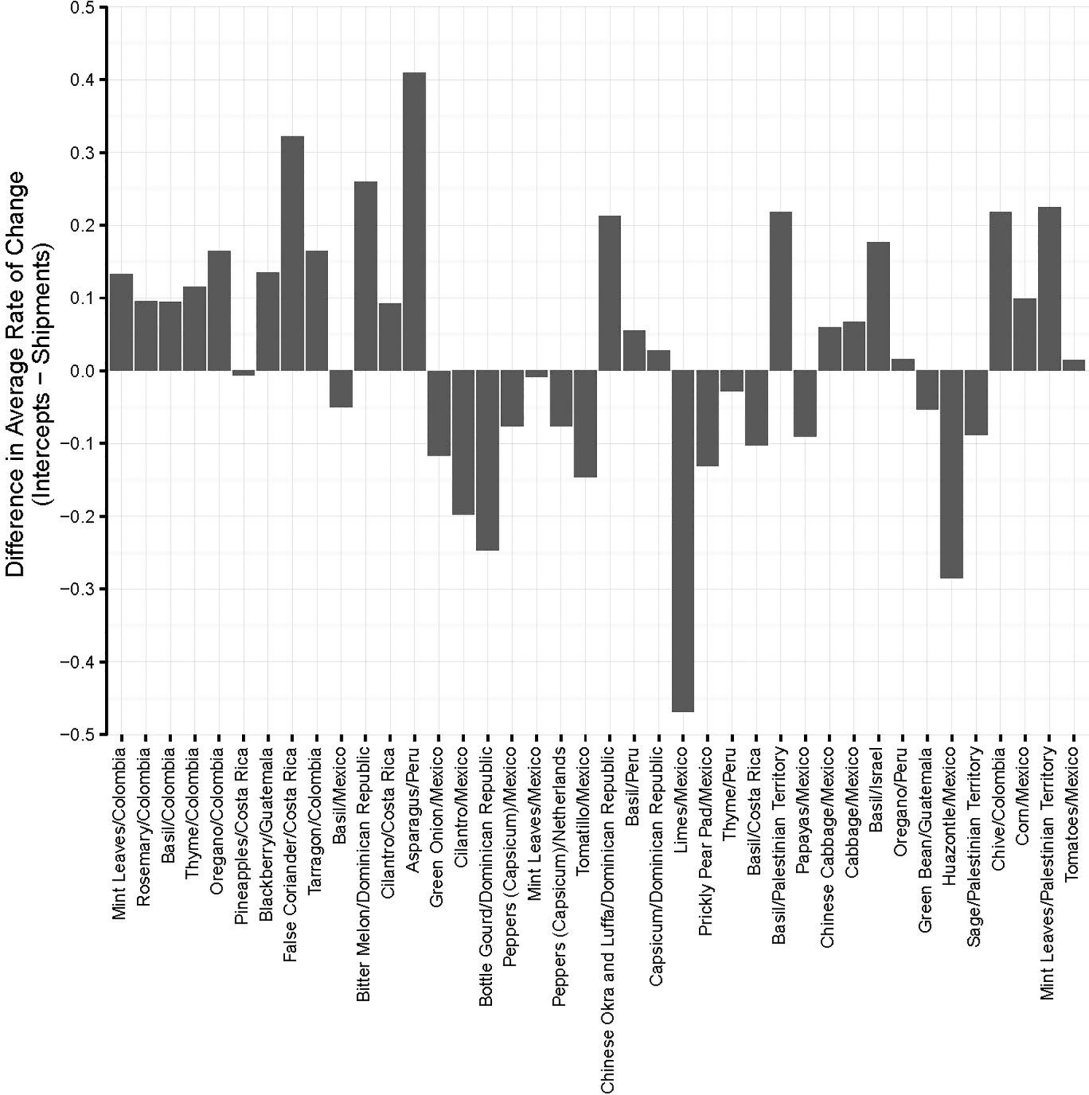
Growth rates of actionable pest detections and shipments.

Increases in propagule pressure associated with the growth of imports can strain the capacity of a country’s phytosanitary system and thus heighten the importance of directing resources toward the greatest threats. Our model helps identify those threats. Fig 7 depicts the difference in the growth rates of actionable pest detections and shipments for the 40 commodity/country combinations with the greatest expected number of annual arrivals. Growth in actionable pest arrivals in herbs from Colombia, Costa Rica, and the Palestinian Territories; blackberries from Guatemala; and squashes (bitter melon, luffa) from the Dominican Republic all outstripped growth in shipments, indicating substantial growth in arrivals per shipment and thus worsening of phytosanitary status. On the other side of the spectrum, native Mexican vegetables (prickly pear pads, huazontle) and commodities like green onions and cilantro from Mexico had growth rates in arrivals below growth rates in shipments, indicating reductions in arrivals per shipment and thus improvements in phytosanitary status. Commodities like limes from Mexico, basil from Costa Rica, and green beans from Guatemala had negative growth rates in arrivals that more than offset growth in shipments.

Comparison of growth rates of actionable pest detections and shipments for the top 40 commodity/country of origin combinations with the greatest expected number of annual actionable pest arrivals.

Two complementary courses of action can be taken in cases where arrivals increase faster than shipments: (1) provision of technical assistance to exporters and (2) greater targeting of inspections.

First, APHIS can and does provide technical assistance to exporters and exporting country governments to help develop and implement protocols that reduce actionable pest presence. APHIS maintains offices worldwide with staff working with foreign government officials to resolve sanitary and phytosanitary issues and with industry trade groups and exporting firms directly to help them meet US standards for invasive pests (as well as other sanitary and phytosanitary requirements). There are offices in most Central and South American countries as well as throughout Mexico; there are offices. The APHIS International Technical and Regulatory Capacity Building Center, created in 2007, offers programs to help exporting countries improve their performance in meeting US sanitary and phytosanitary regulations. In the Caribbean, APHIS also works with trading partners on implementing the Caribbean Regional Invasive Species Intervention Strategy in order to reduce inter-country movements of invasive pests in the region. Our estimates can be used to set priorities for technical assistance to exporters and capacity building for exporting country governments by identifying the commodities and countries or origin in both greatest and least need of technical assistance.

Second, APHIS can and does perform enhanced screening of imports with worsening phytosanitary status as a precaution against greater entry rates. Analyses like that conducted in this paper can facilitate cost effective targeting as well as reallocation of resources from low to high need. Our analysis identifies the pathways of greatest phytosanitary concern. It provides quantitative assessments of arrival probabilities that can be used for initial targeting of enhanced inspection effort a suggested in the literature [18–20]. Our estimates can also serve as priors for updating estimates of arrival probabilities, thus making it possible to improve risk management, make better use of available resources, and reduce incentives to circumvent phytosanitary safeguards by learning from experience with future shipments.

## Supporting information

S1 Table Logit model estimated coefficients and odds ratios. (PDF)

S2 Table Estimated probabilities and numbers of expected potential pest arrivals by commodity. (PDF)

S3 Table Estimated probabilities and numbers of expected potential pest arrivals by country or region of origin. (PDF)
