# Supplementary material for: The fruit and vegetable import pathway for potential invasive pest arrivals
Source: PLoS One. 2018 Feb 16;13(2):e0192280. doi: 10.1371/journal.pone.0192280 (PMC5815589; doi:10.1371/journal.pone.0192280)
Supplement: S1 Table — (PDF) [file pone.0192280.s001.pdf]

Table S1. Logit model estimated coefficients and odds ratios

	Coefficient	Standard Error	z	P>|z|	95% Confidence Interval	
Constant	-6.209653	0.1665041	-37.29	0	-6.535995	-5.883311
Log quantity	0.0022848	0.002521	0.91	0.365	-0.0026563	0.0072258
Season (base level = Summer)						
Winter	-0.1318768	0.0072387	-18.22	0	-0.1460644	-0.1176891
Tariff duty free status (base level = positive duty)						
Duty free	0.3544091	0.0178626	19.84	0	0.319399	0.3894192
Commodity (base level = Aloe Vera)						
Amaranth	1.4726	0.1071834	13.74	0	1.262525	1.682676
Apples	0.3884522	0.1045735	3.71	0	0.1834919	0.5934126
Apricots, Peaches and Nectarines	-1.362368	0.1713415	-7.95	0	-1.698191	-1.026544
Arrowroot, Salep, Jerusalem Artichokes	-3.259664	0.2997338	-10.88	0	-3.847132	-2.672197
Artichokes and Cardoons	-1.925792	0.2979389	-6.46	0	-2.509741	-1.341842
Arugula	-1.010263	0.0968679	-10.43	0	-1.200121	-0.8204055
Asparagus	0.5270184	0.0749275	7.03	0	0.3801632	0.6738735
Avocados	-3.17777	0.1007648	-31.54	0	-3.375265	-2.980275
Bamboo Shoots and Water Chestnuts	-1.609433	0.2181447	-7.38	0	-2.036988	-1.181877
Banana, Flower or Leaves	-2.007235	0.0992039	-20.23	0	-2.201672	-1.812799
Bananas	-2.701203	0.0776469	-34.79	0	-2.853388	-2.549018
Basil	0.3196118	0.0714195	4.48	0	0.1796322	0.4595914
Bay Leaves	-1.834232	0.1253074	-14.64	0	-2.07983	-1.588633
Bean Sprouts	-1.285008	0.1483363	-8.66	0	-1.575742	-0.994274
Beans and Legumes Not Elsewhere Specified	-2.744347	0.1529847	-17.94	0	-3.044191	-2.444502
Beet	-1.497179	0.1097123	-13.65	0	-1.712211	-1.282147
Bitter Melon or Bitter Melon Leaves	-0.0155262	0.0744733	-0.21	0.835	-0.1614913	0.1304388
Blackberry	0.0160685	0.0747316	0.22	0.83	-0.1304028	0.1625397
Blueberries, Cranberries, Vaccinium	-1.584174	0.0923718	-17.15	0	-1.765219	-1.403129
Bottle Gourd	0.8890496	0.0806384	11.03	0	0.7310013	1.047098
Breadfruit	-2.720042	0.1246944	-21.81	0	-2.964438	-2.475645
Broccoli	-0.7561337	0.082121	-9.21	0	-0.917088	-0.5951795
Brussels Sprouts	-2.653347	0.1653963	-16.04	0	-2.977518	-2.329176

Cabbage	-0.4940665	0.0787709	-6.27	0	-0.6484546	-0.3396784
Cantaloupes	-3.449333	0.1303473	-26.46	0	-3.704809	-3.193857
Carrots	-2.819707	0.1202489	-23.45	0	-3.05539	-2.584023
Cassava	-2.195134	0.0894169	-24.55	0	-2.370388	-2.019881
Cauliflower	-0.839985	0.1521363	-5.52	0	-1.138167	-0.5418033
Celery	-0.0436671	0.0847933	-0.51	0.607	-0.2098589	0.1225248
Chayote	-1.153082	0.0786041	-14.67	0	-1.307144	-0.9990211
Cherries	-0.969712	0.1071654	-9.05	0	-1.179752	-0.7596717
Chervil	-0.7729101	0.1049204	-7.37	0	-0.9785503	-0.5672699
Chickpeas	-1.595529	0.2214479	-7.2	0	-2.029559	-1.161499
Chicory	-3.079596	0.1267982	-24.29	0	-3.328116	-2.831076
Chili Peppers	-2.232345	0.0935078	-23.87	0	-2.415617	-2.049073
Chinese Cabbage	-0.0975444	0.0801728	-1.22	0.224	-0.2546802	0.0595914
Chinese Kale	-1.209224	0.1199483	-10.08	0	-1.444318	-0.9741298
Chinese Okra and Luffa	-0.8372776	0.0796511	-10.51	0	-0.9933908	-0.6811644
Chive	-1.315215	0.0770206	-17.08	0	-1.466173	-1.164258
Cilantro	-0.2630153	0.0724772	-3.63	0	-0.405068	-0.1209626
Citrus Fruit	-0.5104101	0.1772054	-2.88	0.004	-0.8577264	-0.1630938
Clusterbean	-3.654673	0.1541704	-23.71	0	-3.956842	-3.352505
Coconuts	-2.649725	0.118522	-22.36	0	-2.882024	-2.417426
Corn	-0.8947982	0.0789838	-11.33	0	-1.049604	-0.7399928
Cucumbers	-2.005458	0.0809038	-24.79	0	-2.164027	-1.84689
Currant, Gooseberry, Berries, Tamarinds	-1.337182	0.185194	-7.22	0	-1.700155	-0.9742081
Dasheen or Dasheen Leaves	-1.978232	0.0797839	-24.79	0	-2.134606	-1.821859
Dates & Figs	-0.119619	0.1441095	-0.83	0.407	-0.4020685	0.1628305
Dill	-0.4427992	0.0778309	-5.69	0	-0.5953449	-0.2902535
Durians	-1.173783	0.2319082	-5.06	0	-1.628315	-0.7192515
Eggplants	-1.580801	0.0788051	-20.06	0	-1.735256	-1.426346
Epazote	-0.7446976	0.0892658	-8.34	0	-0.9196553	-0.56974
False Coriander	0.3576518	0.0780755	4.58	0	0.2046267	0.5106769
Fava Bean	-1.200959	0.1082531	-11.09	0	-1.413131	-0.9887865
Fruit Not Elsewhere Specified	-1.853618	0.109759	-16.89	0	-2.068741	-1.638494
Garlic	-1.575249	0.1185887	-13.28	0	-1.807679	-1.34282

Genip	-1.341001	0.1134892	-11.82	0	-1.563436	-1.118567
Ginger	-1.502307	0.0959387	-15.66	0	-1.690343	-1.314271
Gourds Not Elsewhere Specified	-0.8761714	0.1610769	-5.44	0	-1.191876	-0.5604665
Grapes	-1.480738	0.1004294	-14.74	0	-1.677576	-1.2839
Green Bean	-1.091568	0.0771833	-14.14	0	-1.242845	-0.9402915
Green Onion	-0.0897368	0.0745709	-1.2	0.229	-0.2358932	0.0564195
Guavas, Mangoes, Mangosteens	-1.375034	0.0880478	-15.62	0	-1.547605	-1.202464
Honeydews and Other Melons	-1.959606	0.1678108	-11.68	0	-2.288509	-1.630703
Huazontle	0.966812	0.0832981	11.61	0	0.8035506	1.130073
Ivy Gourd	-1.519876	0.0929941	-16.34	0	-1.702141	-1.337611
Jackfruit	-2.221781	0.161318	-13.77	0	-2.537959	-1.905604
Jicama	-2.100815	0.1042284	-20.16	0	-2.305099	-1.896531
Kale	-0.5123095	0.1088217	-4.71	0	-0.7255961	-0.2990229
Kiwi	-2.366385	0.1468508	-16.11	0	-2.654207	-2.078562
Kohlrabi, Kale, Brassicas Not Elsewhere Specified	-1.261884	0.1420701	-8.88	0	-1.540336	-0.9834314
Kola Nut	-0.2436214	0.368346	-0.66	0.508	-0.9655664	0.4783235
Lambsquarter	-0.7499973	0.1253949	-5.98	0	-0.9957668	-0.5042279
Leek	-1.709045	0.0970499	-17.61	0	-1.899259	-1.518831
Lemongrass	-1.522089	0.193448	-7.87	0	-1.90124	-1.142938
Lemons	-0.5481479	0.0946317	-5.79	0	-0.7336226	-0.3626732
Lettuce, Head or Leaf	-0.1892093	0.0772132	-2.45	0.014	-0.3405445	-0.0378742
Limes	0.0922553	0.0756902	1.22	0.223	-0.0560948	0.2406053
Loroco	-0.9288679	0.136001	-6.83	0	-1.195425	-0.6623108
Maguey or Maguey Leaves	-1.351879	0.1255291	-10.77	0	-1.597912	-1.105847
Mandarins, Clementines, Citrus Hybrids	-2.748468	0.1281653	-21.44	0	-2.999667	-2.497268
Marjoram	0.0963265	0.0773522	1.25	0.213	-0.055281	0.2479341
Mint Leaves	0.1993513	0.0722362	2.76	0.006	0.057771	0.3409317
Mizuna	-0.9962925	0.2070735	-4.81	0	-1.402149	-0.5904359
Mushrooms	-2.122219	0.1287368	-16.48	0	-2.374539	-1.8699
Mustard or Mustard Greens	-0.9564231	0.1176608	-8.13	0	-1.187034	-0.7258121
Nuts	-1.506561	0.1935873	-7.78	0	-1.885985	-1.127137
Okra	-0.9099366	0.081404	-11.18	0	-1.069486	-0.7503876
Olives	0.7389786	0.2138709	3.46	0.001	0.3197994	1.158158

Onions	-2.224564	0.0935546	-23.78	0	-2.407927	-2.0412
Oranges	-1.887484	0.105628	-17.87	0	-2.094511	-1.680457
Oregano	0.0949294	0.073486	1.29	0.196	-0.0491006	0.2389593
Pak Choi	-0.7636786	0.1517183	-5.03	0	-1.061041	-0.4663162
Papalo	-1.597194	0.1560943	-10.23	0	-1.903133	-1.291255
Papayas	-1.049841	0.0760208	-13.81	0	-1.198839	-0.900843
Parsley	-0.5100875	0.0854197	-5.97	0	-0.677507	-0.3426679
Pears and Quinces	-0.8888831	0.1333904	-6.66	0	-1.150324	-0.6274427
Peas	-0.9131623	0.0829396	-11.01	0	-1.075721	-0.7506038
Pepicha	-1.51269	0.1527842	-9.9	0	-1.812142	-1.213238
Peppers (Capsicum)	-1.541719	0.0720381	-21.4	0	-1.682911	-1.400527
Peppers (Piper)	0.2134961	0.1265317	1.69	0.092	-0.0345014	0.4614936
Pineapples	-1.59134	0.0735833	-21.63	0	-1.73556	-1.447119
Plant and Plant Parts Not Elsewhere Specified	-0.381146	0.1836821	-2.08	0.038	-0.7411563	-0.0211358
Plantains	-2.825222	0.0813081	-34.75	0	-2.984583	-2.665861
Plums, Prunes, Sloes	0.0636953	0.1313255	0.49	0.628	-0.1936981	0.3210886
Pointed Gourd	-1.112181	0.0986786	-11.27	0	-1.305587	-0.9187742
Prickly Pear Fruit	-1.257761	0.0920052	-13.67	0	-1.438088	-1.077434
Prickly Pear Pad	-1.089057	0.0796574	-13.67	0	-1.245183	-0.9329319
Pumpkin	-1.666058	0.0942972	-17.67	0	-1.850877	-1.481239
Purslane	-0.7991427	0.0914956	-8.73	0	-0.9784708	-0.6198146
Radishes	-0.8872881	0.0816606	-10.87	0	-1.04734	-0.7272362
Rambutan	-0.3815596	0.0935468	-4.08	0	-0.564908	-0.1982112
Raspberry	-1.544101	0.161323	-9.57	0	-1.860288	-1.227913
Rhubarb	-1.839784	0.1598069	-11.51	0	-2.153	-1.526568
Rosemary	0.2731557	0.0729195	3.75	0	0.1302361	0.4160753
Sage	0.4694569	0.0763198	6.15	0	0.3198729	0.6190409
Salsify, Celeriac, Edible Roots Not Elsewhere Specified	-3.080093	0.1635308	-18.83	0	-3.400608	-2.759579
Savory	-0.7863086	0.1332605	-5.9	0	-1.047494	-0.5251228
Shallot	-1.571439	0.1228029	-12.8	0	-1.812128	-1.33075
Snow Pea Sprouts	-1.195116	0.1296904	-9.22	0	-1.449304	-0.9409272
Sorrel	-1.072709	0.1159443	-9.25	0	-1.299955	-0.8454619
Spices Not Elsewhere Specified	-1.3911	0.268256	-5.19	0	-1.916872	-0.8653274

Spinach	-0.7693756	0.0956894	-8.04	0	-0.9569234	-0.5818278
Squash	-1.774889	0.075067	-23.64	0	-1.922018	-1.627761
Strawberries	-0.3361071	0.1159389	-2.9	0.004	-0.5633431	-0.1088711
Sweet Potatoes	-1.462096	0.1242031	-11.77	0	-1.705529	-1.218662
Swiss Chard	-1.018703	0.1239809	-8.22	0	-1.261701	-0.7757051
Tahitian and Persian Limes	-0.9186821	0.0871109	-10.55	0	-1.089416	-0.7479478
Tarragon	-0.1033748	0.0750206	-1.38	0.168	-0.2504126	0.0436629
Tepeguaje	-0.1087254	0.0838755	-1.3	0.195	-0.2731184	0.0556676
Thyme	0.023538	0.0727481	0.32	0.746	-0.1190457	0.1661216
Tomatillo and Cape Gooseberry	-1.059801	0.0773565	-13.7	0	-1.211417	-0.9081856
Tomatoes	-1.226403	0.0756703	-16.21	0	-1.374714	-1.078092
Truffles	-3.925384	0.2803407	-14	0	-4.474842	-3.375927
Vegetables Not Elsewhere Specified	-2.48318	0.1545752	-16.06	0	-2.786142	-2.180218
Watermelons	-1.372129	0.0982709	-13.96	0	-1.564736	-1.179522
Yams	-1.099158	0.134069	-8.2	0	-1.361928	-0.8363878
Yard-Long Bean	-1.686021	0.1059453	-15.91	0	-1.89367	-1.478372
Country/region (base level = Canada)						
Africa Secondary	3.790262	0.3080644	12.3	0	3.186467	4.394057
Antigua and Barbuda	3.603942	0.5306917	6.79	0	2.563806	4.644079
Argentina	3.762504	0.1596462	23.57	0	3.449603	4.075404
Australia	3.682153	0.1987185	18.53	0	3.292672	4.071634
Belgium	3.374396	0.1689671	19.97	0	3.043226	3.705565
Belize	1.898804	0.2468545	7.69	0	1.414978	2.38263
Brazil	3.006041	0.159951	18.79	0	2.692543	3.319539
Bulgaria	4.143544	0.2382212	17.39	0	3.676639	4.610449
Caribbean Secondary	4.854235	0.5058251	9.6	0	3.862836	5.845634
Chile	2.90726	0.1584651	18.35	0	2.596674	3.217846
China	2.010608	0.1700723	11.82	0	1.677272	2.343943
Colombia	3.639555	0.1495477	24.34	0	3.346447	3.932663
Costa Rica	4.233395	0.1500652	28.21	0	3.939273	4.527518
Croatia	3.569602	0.5834346	6.12	0	2.426091	4.713113
Dominica	2.763855	0.1713995	16.13	0	2.427918	3.099792
Dominican Republic	3.286455	0.1497617	21.94	0	2.992927	3.579982

Ecuador	3.523317	0.1518717	23.2	0	3.225654	3.82098
El Salvador	2.633123	0.1989166	13.24	0	2.243254	3.022992
Europe Secondary	2.896469	0.2981372	9.72	0	2.312131	3.480807
Fiji	2.497898	0.3417004	7.31	0	1.828178	3.167619
France	2.195004	0.2110508	10.4	0	1.781352	2.608656
Germany	1.763816	0.6159465	2.86	0.004	0.5565829	2.971049
Ghana	3.267955	0.2740551	11.92	0	2.730817	3.805094
Grenada	3.325688	0.2135191	15.58	0	2.907198	3.744177
Guatemala	3.5456	0.1501338	23.62	0	3.251343	3.839857
Guyana	4.120061	0.1665244	24.74	0	3.793679	4.446443
Haiti	2.16871	0.3199403	6.78	0	1.541638	2.795782
Honduras	3.526623	0.1532914	23.01	0	3.226177	3.827068
Hungary	4.029443	0.5363287	7.51	0	2.978258	5.080628
India	2.299388	0.3047794	7.54	0	1.702032	2.896745
Israel	2.114503	0.1498818	14.11	0	1.820741	2.408266
Italy	3.459706	0.1929288	17.93	0	3.081573	3.83784
Jamaica	2.104874	0.1659492	12.68	0	1.77962	2.430129
Japan	1.924526	0.2227873	8.64	0	1.487871	2.361181
Mexico	1.35538	0.149322	9.08	0	1.062715	1.648046
Middle East Secondary	5.275925	0.479645	11	0	4.335838	6.216012
Morocco	2.074918	0.2819382	7.36	0	1.522329	2.627507
Netherlands	3.000858	0.151341	19.83	0	2.704235	3.297481
New Zealand	4.020688	0.1611181	24.95	0	3.704902	4.336474
Nicaragua	3.694253	0.1604811	23.02	0	3.379716	4.00879
Nigeria	5.382136	0.3688852	14.59	0	4.659134	6.105137
Palestinian Territory	2.756373	0.1501903	18.35	0	2.462006	3.050741
Panama	4.489308	0.1617701	27.75	0	4.172244	4.806371
Peru	3.711293	0.1499663	24.75	0	3.417364	4.005221
Poland	2.403041	0.7295798	3.29	0.001	0.973091	3.832991
Portugal	3.545871	0.530779	6.68	0	2.505564	4.586179
SE Asia & Oceania Secondary	4.208041	0.394885	10.66	0	3.434081	4.982002
South Africa	3.346013	0.1815533	18.43	0	2.990175	3.701851
South America Secondary	3.8245	1.083352	3.53	0	1.701169	5.947832

South Korea	3.495611	0.1919474	18.21	0	3.119401	3.871821
Spain	3.016084	0.1642231	18.37	0	2.694213	3.337956
St. Kitts and Nevis	3.39755	0.4414126	7.7	0	2.532398	4.262703
St. Lucia	3.814319	0.2908848	13.11	0	3.244195	4.384442
St. Vincent and the Grenadines	2.751947	0.3411118	8.07	0	2.08338	3.420514
Taiwan	2.622223	0.4120632	6.36	0	1.814594	3.429853
Thailand	3.155905	0.205665	15.34	0	2.752809	3.559001
Trinidad and Tobago	3.577742	0.1619985	22.09	0	3.260231	3.895254
Turkey	2.644273	0.3916947	6.75	0	1.876565	3.41198
Ukraine	4.070821	0.655269	6.21	0	2.786517	5.355125
United Kingdom	5.443278	0.3271912	16.64	0	4.801995	6.084561
Uruguay	1.883046	0.6026522	3.12	0.002	0.701869	3.064222
Venezuela	3.346608	0.5404585	6.19	0	2.287328	4.405887
Viet Nam	2.627596	0.47924	5.48	0	1.688303	3.566889
Region of entry (base level = Southeast)						
South	1.809541	0.0180569	100.21	0	1.77415	1.844932
West	0.5156468	0.0179436	28.74	0	0.4804779	0.5508156
Northeast	1.197552	0.0117958	101.52	0	1.174432	1.220671
Middle	0.7596824	0.0420633	18.06	0	0.6772398	0.842125
Number of observations	2,759,224					
Mean Intercept	0.0328524					
