# Supplementary material for: The fruit and vegetable import pathway for potential invasive pest arrivals
Source: PLoS One. 2018 Feb 16;13(2):e0192280. doi: 10.1371/journal.pone.0192280 (PMC5815589; doi:10.1371/journal.pone.0192280)
Supplement: S2 Table — (PDF) [file pone.0192280.s002.pdf]

Table S3. Estimated Probabilities and Numbers of Expected Potential Pest Arrivals by Commodity

Commodity	Probability of Intercept	Likelihood Relative to Sample Average	Average Annual Shipments	Expected Number of Annual Potential Pest Arrivals	Share of Annual Shipments	Share of Expected Arrivals
Basil	0.0932	2.84	8,799.1	820.0	3.19%	8.61%
Rosemary	0.1095	3.33	4,889.1	535.6	1.77%	5.62%
Thyme	0.0936	2.85	5,561.6	520.4	2.02%	5.46%
Chili Peppers	0.0150	0.46	24,262.6	363.7	8.79%	3.82%
False Coriander	0.2917	8.88	1,240.8	361.9	0.45%	3.80%
Cilantro	0.0602	1.83	5,628.0	338.7	2.04%	3.56%
Kola Nut	0.5600	17.05	588.5	329.6	0.21%	3.46%
Pineapples	0.0455	1.38	6,633.0	301.6	2.40%	3.17%
Dates & Figs	0.0744	2.26	3,983.7	296.2	1.44%	3.11%
Citrus Fruit	0.0552	1.68	5,078.3	280.4	1.84%	2.94%
Tarragon	0.0625	1.90	3,734.1	233.3	1.35%	2.45%
Blackberry	0.0865	2.63	2,537.5	219.5	0.92%	2.30%
Asparagus	0.1545	4.70	1,382.4	213.6	0.50%	2.24%
Bitter Melon or Bitter Melon Leaves	0.1017	3.09	2,044.7	207.9	0.74%	2.18%
Parsley	0.0260	0.79	7,056.7	183.2	2.56%	1.92%
Mint Leaves	0.0963	2.93	1,891.4	182.2	0.69%	1.91%
Currant, Gooseberry, Berries, Tamarinds	0.0253	0.77	7,084.4	178.9	2.57%	1.88%
Sage	0.0860	2.62	1,981.9	170.4	0.72%	1.79%
Green Onion	0.0328	1.00	4,754.0	156.1	1.72%	1.64%
Squash	0.0112	0.34	11,781.9	132.0	4.27%	1.39%
Tomatoes	0.0167	0.51	7,427.6	124.0	2.69%	1.30%
Mizuna	0.0191	0.58	6,489.2	123.8	2.35%	1.30%
Oregano	0.0914	2.78	1,335.4	122.1	0.48%	1.28%

Loroco	0.0510	1.55	2,352.4	120.1	0.85%	1.26%
Bottle Gourd	0.3553	10.81	320.6	113.9	0.12%	1.20%
Epazote	0.0264	0.80	3,664.8	96.6	1.33%	1.01%
Bananas	0.0131	0.40	7,347.7	96.2	2.66%	1.01%
Limes	0.0496	1.51	1,775.9	88.1	0.64%	0.92%
Tomatillo and Cape Gooseberry	0.0118	0.36	7,211.4	84.8	2.61%	0.89%
Chayote	0.0226	0.69	3,746.6	84.8	1.36%	0.89%
Chinese Cabbage	0.0469	1.43	1,801.1	84.5	0.65%	0.89%
Guavas, Mangoes, Mangosteens	0.0197	0.60	4,107.1	81.0	1.49%	0.85%
Marjoram	0.0769	2.34	1,020.1	78.4	0.37%	0.82%
Olives	0.0409	1.25	1,795.5	73.5	0.65%	0.77%
Plantains	0.0131	0.40	5,565.4	73.0	2.02%	0.77%
Durians	0.0327	1.00	2,218.3	72.5	0.80%	0.76%
Cabbage	0.0377	1.15	1,920.2	72.3	0.70%	0.76%
Kale	0.0173	0.53	4,073.7	70.5	1.48%	0.74%
Corn	0.0231	0.70	2,904.6	67.1	1.05%	0.70%
Prickly Pear Pad	0.0123	0.37	5,325.0	65.3	1.93%	0.69%
Blueberries, Cranberries, Vaccinium	0.0193	0.59	3,340.4	64.6	1.21%	0.68%
Pak Choi	0.0132	0.40	4,461.2	58.9	1.62%	0.62%
Radishes	0.0193	0.59	2,850.9	55.0	1.03%	0.58%
Broccoli	0.0282	0.86	1,834.5	51.7	0.66%	0.54%
Pears and Quinces	0.0304	0.93	1,671.9	50.8	0.61%	0.53%
Gourds Not Elsewhere Specified	0.0317	0.96	1,594.4	50.5	0.58%	0.53%
Chinese Okra and Luffa	0.0649	1.98	751.2	48.7	0.27%	0.51%
Tepeguaje	0.0426	1.30	1,120.8	47.8	0.41%	0.50%
Lemongrass	0.0239	0.73	1,896.2	45.3	0.69%	0.48%
Celery	0.0514	1.56	859.9	44.2	0.31%	0.46%
Genip	0.0348	1.06	1,265.9	44.1	0.46%	0.46%
Huazontle	0.1234	3.76	348.5	43.0	0.13%	0.45%
Cassava	0.0287	0.88	1,262.7	36.3	0.46%	0.38%

Tahitian and Persian Limes	0.0177	0.54	2,045.4	36.2	0.74%	0.38%
Oranges	0.0177	0.54	1,946.1	34.4	0.71%	0.36%
Apples	0.1420	4.32	240.2	34.1	0.09%	0.36%
Rambutan	0.0722	2.20	414.1	29.9	0.15%	0.31%
Purslane	0.0227	0.69	1,274.9	28.9	0.46%	0.30%
Lettuce, Head or Leaf	0.0553	1.68	520.6	28.8	0.19%	0.30%
Cucumbers	0.0088	0.27	3,150.4	27.8	1.14%	0.29%
Prickly Pear Fruit	0.0129	0.39	2,149.8	27.7	0.78%	0.29%
Cherries	0.0288	0.88	959.2	27.6	0.35%	0.29%
Pumpkin	0.0284	0.86	973.4	27.6	0.35%	0.29%
Chive	0.0202	0.61	1,343.8	27.1	0.49%	0.28%
Mustard or Mustard Greens	0.0183	0.56	1,414.8	25.9	0.51%	0.27%
Amaranth	0.1998	6.08	122.1	24.4	0.04%	0.26%
Arugula	0.0260	0.79	932.3	24.2	0.34%	0.25%
Pointed Gourd	0.0717	2.18	329.0	23.6	0.12%	0.25%
Spinach	0.0200	0.61	1,180.2	23.6	0.43%	0.25%
Honeydews and Other Melons	0.0126	0.38	1,865.0	23.5	0.68%	0.25%
Aloe Vera	0.0645	1.96	352.1	22.7	0.13%	0.24%
Watermelons	0.0189	0.57	1,127.6	21.3	0.41%	0.22%
Pepicha	0.0127	0.39	1,658.7	21.1	0.60%	0.22%
Ivy Gourd	0.0467	1.42	450.4	21.1	0.16%	0.22%
Banana, Flower or Leaves	0.0094	0.29	2,184.9	20.5	0.79%	0.22%
Kiwi	0.0230	0.70	843.3	19.4	0.31%	0.20%
Chinese Kale	0.0141	0.43	1,347.0	19.0	0.49%	0.20%
Avocados	0.0031	0.10	5,943.4	18.7	2.15%	0.20%
Chervil	0.0372	1.13	486.9	18.1	0.18%	0.19%
Fruit Not Elsewhere Specified	0.0164	0.50	1,017.1	16.6	0.37%	0.17%
Yard-Long Bean	0.0155	0.47	1,042.9	16.2	0.38%	0.17%
Beet	0.0083	0.25	1,692.3	14.1	0.61%	0.15%
Strawberries	0.0570	1.74	234.9	13.4	0.09%	0.14%

Sorrel	0.0268	0.82	473.7	12.7	0.17%	0.13%
Plums, Prunes, Sloes	0.0819	2.49	142.9	11.7	0.05%	0.12%
Peas	0.0326	0.99	352.0	11.5	0.13%	0.12%
Garlic	0.0107	0.33	1,033.3	11.1	0.37%	0.12%
Yams	0.0177	0.54	622.3	11.0	0.23%	0.12%
Nuts	0.0175	0.53	618.0	10.8	0.22%	0.11%
Carrots	0.0035	0.11	2,996.2	10.5	1.09%	0.11%
Shallot	0.0143	0.43	715.2	10.2	0.26%	0.11%
Breadfruit	0.0090	0.27	1,114.5	10.0	0.40%	0.10%
Sweet Potatoes	0.0154	0.47	640.9	9.9	0.23%	0.10%
Green Bean	0.0221	0.67	445.8	9.8	0.16%	0.10%
Bay Leaves	0.0156	0.48	626.9	9.8	0.23%	0.10%
Swiss Chard	0.0173	0.53	560.6	9.7	0.20%	0.10%
Peppers (Piper)	0.0726	2.21	133.7	9.7	0.05%	0.10%
Chicory	0.0050	0.15	1,882.9	9.5	0.68%	0.10%
Fava Bean	0.0146	0.44	595.9	8.7	0.22%	0.09%
Cantaloupes	0.0047	0.14	1,843.0	8.6	0.67%	0.09%
Snow Pea Sprouts	0.0156	0.47	544.8	8.5	0.20%	0.09%
Savory	0.0340	1.04	249.8	8.5	0.09%	0.09%
Papayas	0.0184	0.56	442.9	8.1	0.16%	0.09%
Grapes	0.0485	1.47	161.0	7.8	0.06%	0.08%
Ginger	0.0189	0.58	387.9	7.3	0.14%	0.08%
Lemons	0.0546	1.66	133.9	7.3	0.05%	0.08%
Okra	0.0369	1.12	183.1	6.8	0.07%	0.07%
Bean Sprouts	0.0731	2.23	91.6	6.7	0.03%	0.07%
Coconuts	0.0042	0.13	1,443.6	6.0	0.52%	0.06%
Peppers (Capsicum)	0.0138	0.42	432.1	6.0	0.16%	0.06%
Kohlrabi, Kale, Brassicas Not Elsewhere Specified	0.0112	0.34	500.9	5.6	0.18%	0.06%
Cauliflower	0.0243	0.74	230.4	5.6	0.08%	0.06%
Vegetables Not Elsewhere Specified	0.0072	0.22	765.9	5.5	0.28%	0.06%

Beans and Legumes Not Elsewhere Specified	0.0094	0.29	582.6	5.5	0.21%	0.06%
Dill	0.0530	1.61	102.2	5.4	0.04%	0.06%
Mandarins, Clementines, Citrus Hybrids	0.0095	0.29	567.6	5.4	0.21%	0.06%
Rhubarb	0.0165	0.50	308.3	5.1	0.11%	0.05%
Raspberry	0.0121	0.37	412.8	5.0	0.15%	0.05%
Papalo	0.0117	0.36	424.2	5.0	0.15%	0.05%
Salsify, Celeriac, Edible Roots Not Elsewhere Specified	0.0045	0.14	1,038.8	4.7	0.38%	0.05%
Apricots, Peaches and Nectarines	0.0220	0.67	213.8	4.7	0.08%	0.05%
Brussels Sprouts	0.0029	0.09	1,563.7	4.5	0.57%	0.05%
Jackfruit	0.0068	0.21	639.7	4.3	0.23%	0.05%
Leek	0.0126	0.38	339.8	4.3	0.12%	0.04%
Plant and Plant Parts Not Elsewhere Specified	0.0471	1.43	87.0	4.1	0.03%	0.04%
Maguey or Maguey Leaves	0.0164	0.50	231.2	3.8	0.08%	0.04%
Jicama	0.0041	0.12	708.9	2.9	0.26%	0.03%
Dasheen or Dasheen Leaves	0.0191	0.58	146.5	2.8	0.05%	0.03%
Eggplants	0.0240	0.73	110.1	2.6	0.04%	0.03%
Truffles	0.0046	0.14	566.5	2.6	0.21%	0.03%
Bamboo Shoots and Water Chestnuts	0.0116	0.35	215.2	2.5	0.08%	0.03%
Chickpeas	0.0088	0.27	262.4	2.3	0.10%	0.02%
Spices Not Elsewhere Specified	0.0122	0.37	147.3	1.8	0.05%	0.02%
Mushrooms	0.0103	0.31	141.5	1.5	0.05%	0.02%
Artichokes and Cardoons	0.0046	0.14	261.0	1.2	0.09%	0.01%
Arrowroot, Salep, Jerusalem Artichokes	0.0024	0.07	507.5	1.2	0.18%	0.01%
Onions	0.0140	0.43	63.5	0.9	0.02%	0.01%
Lambsquarter	0.0280	0.85	12.5	0.3	0.005%	0.004%
Clusterbean	0.0037	0.11	81.5	0.3	0.03%	0.003%
