# Supplementary material for: The fruit and vegetable import pathway for potential invasive pest arrivals
Source: PLoS One. 2018 Feb 16;13(2):e0192280. doi: 10.1371/journal.pone.0192280 (PMC5815589; doi:10.1371/journal.pone.0192280)
Supplement: S3 Table — (PDF) [file pone.0192280.s003.pdf]

Table S4. Estimated Probabilities and Numbers of Expected Potential Pest Arrivals by Country or Region of Origin

Country/Region	Probability of Intercept	Likelihood Relative to Sample Average	Average Annual Shipments	Expected Number of Annual Potential Pest Arrivals	Share of Annual Shipments	Share of Expected Arrivals
Mexico	0.0164	0.50	146,740.6	2413.4	53.18%	26.62%
Colombia	0.1068	3.25	18,675.5	1993.9	6.77%	21.99%
Costa Rica	0.0865	2.63	9,907.4	856.9	3.59%	9.45%
Dominican Republic	0.0367	1.12	21,822.1	799.8	7.91%	8.82%
Peru	0.0771	2.35	7,511.5	579.5	2.72%	6.39%
Guatemala	0.0392	1.19	13,289.6	521.5	4.82%	5.75%
Israel	0.0556	1.69	7,710.5	428.5	2.79%	4.73%
Palestinian Territory	0.1090	3.32	3,592.3	391.6	1.30%	4.32%
Netherlands	0.0214	0.65	10,939.4	234.0	3.96%	2.58%
Ecuador	0.0267	0.81	6,365.0	170.0	2.31%	1.88%
Honduras	0.0234	0.71	4,147.5	96.9	1.50%	1.07%
Chile	0.0236	0.72	3,604.6	84.9	1.31%	0.94%
Argentina	0.0412	1.25	1,550.0	63.9	0.56%	0.70%
New Zealand	0.0684	2.08	678.5	46.4	0.25%	0.51%
Brazil	0.0365	1.11	1,027.0	37.5	0.37%	0.41%
Nicaragua	0.0304	0.93	1,152.8	35.1	0.42%	0.39%
Panama	0.0416	1.27	752.4	31.3	0.27%	0.35%
Jamaica	0.0177	0.54	1,566.2	27.7	0.57%	0.31%
Trinidad and Tobago	0.0424	1.29	650.7	27.6	0.24%	0.30%
Guyana	0.1524	4.64	167.3	25.5	0.06%	0.28%
Spain	0.0240	0.73	1,034.4	24.8	0.37%	0.27%
China	0.0072	0.22	3,188.2	23.0	1.16%	0.25%
Belgium	0.0215	0.66	822.0	17.7	0.30%	0.20%
Dominica	0.0110	0.33	1,399.7	15.3	0.51%	0.17%

Italy	0.0204	0.62	665.2	13.6	0.24%	0.15%
South Africa	0.0368	1.12	307.9	11.3	0.11%	0.12%
Thailand	0.0190	0.58	498.8	9.5	0.18%	0.10%
Korea, South	0.0388	1.18	234.8	9.1	0.09%	0.10%
El Salvador	0.0313	0.95	284.2	8.9	0.10%	0.10%
Australia	0.0242	0.74	342.9	8.3	0.12%	0.09%
Nigeria	0.5588	17.01	13.6	7.6	0.005%	0.08%
France	0.0150	0.46	325.6	4.9	0.12%	0.05%
Grenada	0.0177	0.54	265.3	4.7	0.10%	0.05%
Canada	0.0023	0.07	1,998.9	4.6	0.72%	0.05%
Bulgaria	0.0423	1.29	103.9	4.4	0.04%	0.05%
Japan	0.0058	0.18	693.3	4.0	0.25%	0.04%
Belize	0.0106	0.32	254.2	2.7	0.09%	0.03%
Ghana	0.0708	2.16	36.7	2.6	0.01%	0.03%
Morocco	0.0065	0.20	309.2	2.0	0.11%	0.02%
St. Lucia	0.0215	0.65	79.1	1.7	0.03%	0.02%
United Kingdom of Great	0.1650	5.02	10.3	1.7	0.004%	0.02%
Africa Secondary	0.1024	3.12	16.6	1.7	0.01%	0.02%
Europe Secondary	0.0202	0.62	84.1	1.7	0.03%	0.02%
India	0.0137	0.42	109.3	1.5	0.04%	0.02%
Haiti	0.0070	0.21	184.4	1.3	0.07%	0.01%
Fiji	0.0102	0.31	117.2	1.2	0.04%	0.01%
Caribbean Secondary	0.0820	2.50	14.3	1.2	0.01%	0.01%
St. Vincent and the Gren	0.0082	0.25	133.8	1.1	0.05%	0.01%
Turkey	0.0166	0.51	48.2	0.8	0.02%	0.01%
Taiwan	0.0056	0.17	124.7	0.7	0.05%	0.01%
St. Kitts and Nevis	0.0204	0.62	29.4	0.6	0.01%	0.01%
Southeast Asia and Oceania Secondary	0.0600	1.83	10.0	0.6	0.004%	0.01%
Saudi Arabia	0.6250	19.02	0.8	0.5	0.0003%	0.01%
Hungary	0.0086	0.26	58.1	0.5	0.02%	0.01%

Viet Nam	0.0149	0.45	33.6	0.5	0.01%	0.01%
Middle East Secondary	0.3571	10.87	1.4	0.5	0.001%	0.01%
Croatia	0.0052	0.16	77.5	0.4	0.03%	0.004%
Portugal	0.0294	0.90	13.6	0.4	0.005%	0.004%
Antigua and Barbuda	0.0255	0.78	15.7	0.4	0.01%	0.004%
Venezuela	0.0097	0.29	41.4	0.4	0.02%	0.004%
Uruguay	0.0045	0.14	66.3	0.3	0.02%	0.003%
Ukraine	0.0103	0.31	29.1	0.3	0.01%	0.003%
Germany	0.0200	0.61	15.0	0.3	0.01%	0.003%
Poland	0.0159	0.48	12.6	0.2	0.005%	0.002%
